# Knowledge, Attitude and Practice of Pregnant Women towards Varicella and Their Children’s Varicella Vaccination: Evidence from Three Distrcits in Zhejiang Province, China

**DOI:** 10.3390/ijerph14101110

**Published:** 2017-09-24

**Authors:** Yu Hu, Yaping Chen, Ying Wang, Hui Liang

**Affiliations:** Institute of Immunization and Prevention, Zhejiang Center for Disease Control and Prevention, No. 3399 Binsheng Road, Binjiang District, Hangzhou 310051, China; ypchen@cdc.zj.cn (Y.C.); ywang@cdc.zj.cn (Y.W.); hliang@cdc.zj.cn (H.L.)

**Keywords:** varicella, knowledge, attitude, practice, vaccination, pregnant woman, children

## Abstract

*Background*: The objectives of this study were to examine the knowledge, attitudes and practice (KAP) towards varicella and varicella vaccine (VarV) vaccination among pregnant women in three distrcits in Zhejiang Province, China. *Methods*: From 1 January to 31 March 2014, pregnant women with ≥12 gestational weeks were recruited and received a self-administrated questionnaire. The first dose of VarV (VarV1) vaccination status of children from present pregnancy was extracted at 24 months of age from Zhejiang provincial immunization information system (ZJIIS). Three variables was defined as the main outcomes, which included: (1) knowing about both the availability of VarV and the number of doses required; (2) positive attitude towards the utility of varicella vaccination; (3) the vaccination coverage of VarV1, which meant the proportion of children having received the VarV1. Counts and proportions were used to describe the socio-demographic characteristics of study participants, and their relationship with study outcomes were tested using chi-square tests in univariate analysis and logistic regression in multivariable analysis. *Results*: A total of 629 pregnant women participated in this study. The majority of the participants (68.0%) answered correctly about the transmission route of varicella. The proportion of participants who heard about varicella vaccination was 76.5% and 66.8% knew that VarV was currently available. Only 13.5% of the participants answered correctly that the complete VarV series needed two doses. Age, immigration status, education level, household income, and number of children of the pregnant women were significant predictors of the KAP regarding the VarV vaccination. *Conclusions*: The current survey indicated that optimal KAP levels and coverage on VarV vaccination were observed in three districts of Zhejiang Province. Health education programs on varicella and VarV vaccination directed towards both pre-natal and post-natal women are needed, which will result in a better attitude on vaccination of VarV and in a high coverage of VarV.

## 1. Introduction

Varicella is one of the most common and highly contagious viral diseases in children, that can sometimes be fatal due to its serious complications, especially in neonates [1]. The use of varicella vaccine (VarV) has been shown safe and efficacious in reducing the incidence and associated morbidity. Since 1998, VarV has been licensed in Zhejiang Province, located in East China with a population of 70 million (including 20 million migrant people) [2]. The Zhejiang Provincial Center for Disease Control and Prevention (ZJCDC) has recommended a one-dose schedule of VarV for children aged ≥12 months since the approval of VarV [3]. The VarV vaccine is a category II (parent-pay) medicine in China and vaccination is voluntary. After that, the coverage of VarV increased rapidly and a substantial decrease in the incidence of varicella disease, varicella-related morbidity and mortality, and health care costs had been observed in the last decade [3]. ZJCDC updated the VarV recommendation of since January 2014, and the latest recommendation includes a two-dose VarV schedule, with the 1st dose of VarV (VarV1) administered at 12–15 months of age and the 2nd dose of VarV administered at 3–4 years of age, with a minimum interval of 28 days between two doses [3].

According to a previous study conducted in Zhejiang Province, the average coverage of VarV1 was 87.9% among children born in 2013 [3], which was lower than the coverage of vaccines included in the expanded immunization program (EPI). Aside from the reason that VarV is not provided freely, previous studies [4,5] have indicated that mothers play an important and critical role in protecting their children from acquiring and transmitting vaccine-preventable diseases (VPDs) through increasing the vaccination coverage. The specific mechanism is that the knowledge and perception of the benefit of vaccination can influence a mother’s choice to immunize her children. Furthermore, some studies [6,7,8,9] have demonstrated that pregnant womens’ knowledge and attitude regarding vaccination could have an early and positive influence on the practice of getting vaccinated.

To the best that we could ascertain, little is known about knowledge, attitude and practice (KAP) of pregnant women towards varicella in China. Therefore, the aims of this study were to examine the level of knowledge about varicella infection and its vaccination, and the attitudes and practice regarding the vaccination and to get insight into their determinants among pregnant women in Zhejiang Province.

## 2. Methods

### 2.1. Study Setting

This study was conducted in three of the 90 districts in Zhejiang Province, including Yinzhou, Changxing and Kecheng. These three districts were randomly selected according to their socioeconomic development level in 2013, which was categorized by the index of gross domestic product (GDP) per capita. Yinzhou belonged to the high GDP per capita stratum (≥12,000 USD). Changxing was of middle stratum for GDP per capita, at between 10,000 to 12,000 USD. Kecheng was of the low GDP per capita stratum at 8000 to 10,000 USD. The total populations of Yinzhou, Changxing and Kecheng of 2013 were 840,108, 628,175 and 436,856 people, respectively.

### 2.2. Study Subjects

In each district, four obstetric hospitals with an annual number of deliveries ≥500 in 2013 were selected and in total 12 hospitals were chosen as the investigation sites. When more than four hospitals met our selection criteria in a single district, the four with the highest annual number of deliveries were selected. 

Pregnant women with ≥12 gestational weeks were recruited at antenatal classes or at prenatal examinations in participating obstetric hospitals from 1 January 2014, to 31 March 2014. In this study, a migrant was defined as a person who lived in a district other than their hometown (even if from the same province) but had no local registration at their current living place. 

### 2.3. Sample Size

The formula used to estimate the sample size was as follows: $N=\frac{1.96^{2}\times p\times \left(1-p\right)}{d^{2}}$. The coverage of VarV1 among children of the enrolled pregnant women was assumed to be 80% in this study. Furthermore, a *p*-value of 0.05, the desired precision of 5% and a design effect of 2 were also used for the sample size estimation. We used these parameters to estimate the sample size to ensure a larger minimum sample size. Thus, a minimum sample size of 491 subjects would be sufficient to estimate the KAP towards and the coverage of VarV1. Considering the feasibility of this study, the final sample size was 50 eligible pregnant women in each group for every selected hospital or 600 subjects in total.

### 2.4. Enrollment Process

The enrollment period was from 1 January to 31 March 2014. For each hospital, the enrollment would be ended if 50 eligible pregnant women were recruited. Medical staff at each selected hospital approached all pregnant women to determine eligibility. All eligible pregnant women would receive a cover letter describing the study objectives and were asked if they were interested in participating in the study. Pregnant women who expressed interest needed to sign a written informed consent for their participation before getting involved in the research. After that, all participants were required to complete a survey on site, using a self-administered questionnaire.

### 2.5. Questionnaire

A self-administered structured questionnaire was developed by the study team and was pilot-tested among a convenience sample of 20 pregnant women, who were interviewed to verify the general acceptability of the questionnaire in terms of length, clarity, and question formats. The internal reliability was assessed through Cronbach’s α. The final version of questionnaire was structured in four sections. The first section pertained to the questions regarding socio-demographic variables of the respondent. The second section was about the knowledge about varicella, transmission route of the infection, and vaccination. The third section investigated the attitudes towards varicella and vaccination. The fourth section assessed the awareness of information about varicella and vaccination and the source of their information. Each section consisted of questions on a 10-grade scale ranging from 1 (Not worried, Not dangerous, Not useful) to 10 (Much worried, Very dangerous, Very useful) single-choice or multiple-choices. The general internal consistency reliability of the questionnaire evaluated using Cronbach’s α was 0.71. Besides, good internal consistency was found for knowledge, attitude and practice sections with Cronbach’s α coefficient of 0.69, 0.72 and 0.74, respectively.

### 2.6. Vaccination Status

The VarV1 vaccination status of children whose mothers enrolled in this study was extracted at 24 months of age from the Zhejiang Provincial Immunization Information System (ZJIIS). The functions of ZJIIS were previously described elsewhere [10]. 

### 2.7. Outcome

The vaccination coverage was defined as the proportion of children having received the relevant vaccination, independent of their age at vaccination. The outcome variables (dependant variables) included: (1) knowing both the availability of VarV and the number of doses required; (2) positive attitude towards the utility of varicella vaccination; (3) the coverage of VarV1, which was assessed from the ZJIIS data and limited to children from the current pregnancy of study participants. The attitude towards the utility of varicella vaccination was dichotomized into a categorical variable, which was defined as “positive” or “negative” if the corresponding score was greater or equal to or less than the mean value of the original variable. Potential explanatory variables tested at the univariate analysis for the inclusion into the multivariable analysis were the following socio-demographic variables: age of pregnant women, gestational week, immigration status, educational level, monthly household income per capita, occupation, number of children.

### 2.8. Data Analysis

First, descriptive analysis was used to describe the socioeconomic characteristics of study participants and their responses to KAP questions on varicella and VarV vaccination. Second, univariate analyses of chi-square tests was used to detect the associations between three outcomes and the socio-demographic variables. Third, if the specific outcome was significantly associated with each explanatory variable with a *p* ≤ 0.2 in the second step, the variable was included in the logistic regression models. The results of the logistic regression model were presented as odds ratio (OR) with 95% confidence interval (95% CI). All reported *p* values were based on two-tailed tests and were considered statistically significant at level of 0.05 or less. All data were analyzed using the Stata version 14.0 statistical software (Stata Corp. 2015, Stata Statistical Software, College Station, TX, USA).

### 2.9. Ethical Considerations

This study was approved by the Ethical Review Board of Zhejiang Provincial Center for Disease Control and Prevention (T-019-S).

## 3. Results

A total of 690 pregnant women were approached for recruitment, of which 61 (8.8%) pregnant women refused to participate in this study. Finally, 629 pregnant women participated in this study. The demographic characteristics of enrolled pregnant women are presented in Table 1.

The vast majority of the participants had ever heard about varicella (93.0%) and 82.0% of the participants answered correctly the statement that varicella is an infectious disease. 68.0% of them answered correctly the statement that the transmission is mainly person to person by airborne respiratory droplets, direct contact with vesicle fluid of chickenpox cases and patients with herpes zoster. The proportion of participants who had heard about varicella vaccination was 76.5% and 66.8% knew that VarV was currently available, however, only 13.5% of the participants answered correctly that the complete VarV series needed two doses. The results regarding attitude indicated that the concern about infection by varicella was on average 7.9 points on a 1 to 10 scale. The attitude towards causing serious health problems was low, with a mean value of 6.5. The overall perceived utility towards vaccination as a way to protect their child was 7.7 points on a 1 to 10 scale in average (Table 2).

The multivariable logistic regression analysis suggested that older pregnant women, resident pregnant women, women with a higher education level, and better household income were more likely to have a better knowledge and a positive attitude on VarV vaccination, while women with less children had a higher coverage of VarV1. In the three multivariable logistic regression models, the strongest predictors of knowledge, attitude and coverage of VarV1 vaccination were maternal education level, number of children, and immigration status, respectively (Table 3). The R square values for the three logistic regression models were 0.37 for model #1, 0.42 for model #2, and 0.46 for model #3, respectively. The most reported information sources were health care providers (55.8%), family or friends (19.4%), and TV, radio, magazines or newspapers (16.1%).

## 4. Discussion

This study helped to improve the understanding of KAP of pregnant women towards varicella infection and VarV vaccination for their children in Zhejiang Province. Regarding knowledge related to varicella and VarV, the knowledge about VarV was relatively poor since only 66.8% of participants knew that VarV was available and 13.5% of participants correctly indicated the number of doses. This finding was of concern as the VarV had been licenced almost 20 years before. Our results were similar to those of studies conducted among parents from USA and Canada [11,12], with the values of 73.3% and 68%, respectively. Only 26% and 32% of parents had heard about the availability of VarV in UK [13], and Hawaii [14], respectively. However, high level of knowledge on VarV vaccination was observed in Germany [15], where over 95% of parents knew the availability of VarV. We observed a relatively higher coverage of VarV1 but lower knowledge levels on number of recommended VarV doses. One possible reason for this discrepancy was that the knowledge level on dosage for VarV was evaluated during the pregnancy and rather than post-delivery when these women are expected to receive more detailed information on VarV. Since, high levels of knowledge on varicella infection and its vaccination (VarV) among pregnant women can result in wider acceptance of VarV for their children, we suggest that a health education program aimed at increasing the knowledge of varicella infection and VarV vaccination among pregnant women should be implemented. Another potential explanation for why most study participants were wrong about the recommended dosage of VarV, is that the latest schedule of VarV administration was a new recommendation on VarV issued in January 2014. The new recommendation needs sufficient time to get disseminated first among health care workers and the general population later. However, this study was concurrent with the release of new recommendation of VarV, and it would be reasonable that many were uninformed about the updated recommendation of two dose schedule of VarV vaccination.

The VarV1 coverage among children from present pregnancy of participant women was 83.5%, despite not being available free of charge. However, it was much lower than the coverage of any other EPI vaccine [16]. In this study, the coverage of VarV1 was little lower than the coverage estimated based on ZJIIS among children born in 2013, which was 87.9% [3]. The main reason was the coverage value of this study was based on 629 children from four districts while the latter was based on the whole 2013 birth cohort of Zhejiang Province. Another explanation was that the coverage of VarV1 was not even across different areas, even in a same province, as a result of unbalanced performance of immunization programs, health education and different levels of KAP of VarV vaccination. However, the coverage of VarV1 in this study was similar with the value of 87.5% found in Greece [17], and was higher than the results observed in other countries [18,19,20]. A possible explanation of the optimal coverage of VarV1 was the high perceived utility of VarV as a way to protect their child, with a mean value of 7.7 in a 10-point scale. On the aspect of the perceived utility of VarV, similar results had been found in Turkey [21], with a mean value of 3.68 on a 5-point scale and in the US [22] with 4.4 on a 6-point scale. Another possible reason for the high coverage of VarV1 was that 87.8% of the participants stated that they would be willing to vaccinate their children with VarV. In the German survey [15] mentioned above, 83–94% of parents who vaccinated their children believed that VarV was useful, while only 20–30% of parents who did not vaccinate their children believed that VarV was useful.

The results of multivariable analysis indicated several interesting associations. The contributions of the sociodemographic variables to the outcomes of interest indicated that age, immigration status, education level, household income, and number of children of the pregnant women remained in the multivariable analysis model predicting three outcomes of interest. The reasons may be as follows: first, we found older mother were more likely to have a higher KAP level of VarV vaccination and we assumed that the older mothers might have more experience on utilization of health care services, which led to an increased vaccine coverage, including VarV [23]. Second, the association between migrant children and the suboptimal vaccination coverage had been demonstrated [24]. We speculated that the migrants would face the challenges of adapting to the new sociocultural environment while the residents would be better able to avail themselves of the immunization services, as they were familiar with the living areas. Third, maternal education level had been frequently considered as a predictor of childhood vaccination [25,26,27,28,29]. We assumed that a high education level might facilitate a pregnant woman’s communication with physicians, and have a positive influence on completing childhood immunization through better understanding and acceptance of vaccination knowledge in practice. There may be an extra necessity to establish a system that will inform pregnant women about the importance of vaccination aside from using the traditional education methods. Fourth, VarV is still a non-EPI vaccine and involves an out of pocket expense and a lower household income may restrict the expense on parent-pay vaccines like VarV. Blank [30] had demonstrated the importance of a free of charge policy when setting goals of high vaccination coverage. We presume that a high coverage can be expected if VarV were included in EPI. Fifth, our finding was similar to the previous reports from Philippines [31] and the US [32], which indicated that women with less number of children had a higher vaccination coverage. The possible explanation for this association was the allocation of the family resources, which meant that the parental initiative and the dedication of time in addition to the financial expense.

This study indicated that the health care provider was the most frequently consulted information source on varicella and VarV vaccination by the participants, followed by family or friends, and mass media. Since the health care providers have a significantly greater level of knowledge on vaccination compared with other non-professional information sources, the information delivered from a health care provider is effective. Besides, a health care provider is an important and trustworthy source of information for pregnant women regarding childhood vaccination. Our result emphasized the fact that the health care provider was in a unique position for conveying knowledge and in recommending the vaccine. Thus, educating pregnant women regarding the varicella and VarV vaccination to acquire information from health care providers is paramount to encourage them to vaccinate their child. We suggest that health care providers should be aware of their role in communicating with pregnant women regarding VarV vaccination and take advantage of every encounter to inform pregnant women in line with the VarV use recommendation.

Several limitations should be considered. First, due to the on-site nature of the survey, a potential for socially desirable answers might lead to a reporting bias with a tendency to agree with statements when in doubt or to over-reporting acceptability of the vaccination. Second, the data were cross-sectional and obtained from only three districts. Although the associations between the outcomes of interest and certain demographic factors were identified, caution should be taken when interpreting the results owing to the nature of the study design applied, which prevented us from making any conclusion on temporality and causal relationships. Third, the follow-up period of children was only 24 months after birth. A longer observation period might highlight other differences in the coverage of the second dose of VarV. 

## 5. Conclusions

To conclude, the current survey indicated optimal KAP levels and coverage on VarV vaccination were observed in three districts of Zhejiang Province. Health education programs on varicella and VarV vaccination directed towards both pre-natal and post-natal women are needed, which should result in a better attitude toward VarV vaccination and in a high coverage of VarV.

## Figures and Tables

**Table 1:** The socio-demographic information of the participants in this study (*N* = 629).

Sociodemographic	Level	*N*	%
Age (years)	<20	58	9.2
	20–30	386	61.4
	>30	185	29.4
Gestational week	12–21	172	27.3
	22–28	146	23.2
	29–36	225	35.8
	37–42	86	13.7
Immigration status	Migrant	335	53.3
	Resident	294	46.7
Education level	Junior high school or less	54	8.6
	Senior high school or technical school	195	31.0
	College or above	380	60.4
Monthly household income per capita	<800 CNY	128	20.3
	800–1500 CNY	332	52.8
	>1500 CNY	169	26.9
Occupation	No job	54	8.6
	Farmer/worker/businessman	424	67.4
	Civil servants	121	19.2
	Medical staff	30	4.8
Number of children *	0	377	59.9
	1	184	29.3
	≥2	68	10.8

* Excluding the child of the current pregnancy.

**Table 2:** Knowledge, attitude and practice on varicella and its vaccination of the participants in this study (*N* = 629).

Categories	Questions	Positive Response ^#^	
		*N*	%
Knowledge	Have you ever heard about varicella? 1. Yes 2. No	585	93.0
	In your opinion what kind of disease is varicella? 1. Chronic 2. Autoimmune 3. Metabolic 4. Infectious 5. Hereditary 6. Inflammatory	516	82.0
	How can varicella be transmitted? 1. airborne droplets/direct contact/inhalation of aerosols 2. fecal-oral route, contact or ingestion of contaminatedfood or water 3. parenteral or mucosal exposure to body fluids from patients 4. other routes	428	68.0
	Have you ever heard about varicella immunization? 1. Yes 2. No	481	76.5
	Is there a vaccine available for varicella? 1. Yes 2. No	420	66.8
	Do you know the number of doses required for the completion of VarV? 1. Three doses 2. Two doses 3. One dose	85	13.5
	Do you feel you need more information about varicella immunization? 1. Yes 2. No	607	96.5
Practice	Will you vaccinate your child against the varicella in future? 1. Yes 2. No	552	87.8
	Coverage of VarV1 among children at their two years of age	525	83.5
		Mean value	
Attitude	On a scale from 1 to 10, how much are you worried that your child might get varicella in future? *	7.9	
	On a scale from 1 to 10, how serious do you consider varicella? *	6.5	
	On a scale from 1 to 10, how useful do you consider varicella vaccination? *	7.7	

* 1 indicates that you are not worried/serious/useful, 10 indicates that you are very worried/serious/useful; ^#^ for the questions in the knowledge and practice sections. The positive response of each question meant that pregnant woman answered correctly or took a positive practice on varicella and VarV vaccination.

**Table 3:** Univariable and multivariable analysis indicating associations between several variables and outcomes regarding vaccination against varicella (Model#1–Model#3).

Variable ^a^	Level	Model #1		Model #2		Model #3	
		COR (95% *CI*)	AOR (95% *CI*)	COR (95% *CI*)	AOR (95% *CI*)	COR (95% *CI*)	AOR (95% *CI*)
Age (year)	<20	1	1	1	1	1	1
	20–30	1.1 (0.8–1.3)	1.1 (0.9–1.2)	1.3 (0.9–1.4)	1.2 (0.8–1.5)	1.4 (1.2–2.0) *	1.2 (0.9–1.8)
	31–	1.7 (1.4–2.2) *	1.5 (1.2–1.8) *	1.4 (1.1–1.8) *	1.3 (1.1–1.6) *	1.9 (1.4–2.5) *	1.6 (1.3–2.7) *
Immigration status	Migrant	1	1	1	1	1	1
	Resident	2.1 (1.6–2.9) *	2.4 (2.2–3.0) *	1.8 (1.3–2.4) *	1.8 (1.4–2.5) *	1.9 (1.4–2.8) *	2.0 (1.6–3.1) **
Education level	Junior high school or less	1	1	1	1	1	1
	Senior high school or technical school	1.2 (0.7–1.6)	1.1 (0.8–1.4)	1.3 (0.8–1.7)	1.0 (0.6–1.7)	1.2 (0.8–1.7)	1.0 (0.7–1.7)
	College or above	3.4 (2.8–4.1) *	3.2 (2.7–4.0) **	2.7 (2.1–5.2) **	2.4 (1.6–2.9) *	2.1 (1.5–3.0) *	1.9 (1.5–2.9) *
Monthly household income per capita	<800 CNY	1	1	1	1	1	1
	800–1500 CNY	0.9 (0.8–1.1)	1.0 (0.8–1.2)	1.1 (0.8–1.5)	1.2 (0.7–1.8)	1.1 (0.8–1.6)	1.0 (0.7–1.4)
	>1500 CNY	1.9 (1.5–2.4) *	2.1 (1.6–3.3) **	2.4 (1.8–3.3) *	2.2 (1.3–2.5) *	2.1 (1.5–2.9) *	2.0 (1.6–3.3) *
Number of children ^b^	0	1	1	1	1	1	1
	1	1.6 (1.8–2.7) *	1.8 (1.5–2.9) *	2.0 (1.5–2.8) *	1.9 (1.5–3.0) *	0.8 (0.6–1.2)	0.8 (0.6–1.3)
	≥2	2.0 (1.5–2.8) *	2.4 (1.8–3.3) *	2.7 (2.2–5.1) *	2.5 (1.7–4.1) *	0.7 (0.6–0.9) *	0.6 (0.4–0.8) **

Note: ^a^ explanatory variables tested at the univariate analysis for the inclusion into the multivariable analysis were the following: age of pregnant women, gestational week, immigration status, educational level, monthly household income per capita, occupation, number of children. Here only the significant variables were presented. ^b^ excluded the child of the current pregnancy. COR: crude OR, AOR: Adjusted OR. Model #1: knowing both the availability of VarV and the number of doses required; Model #2: positive attitude on the utility of VarV; Model #3: coverage of VarV1 among children whose mothers participated in this study. * *p* < 0.05; ** *p* < 0.01.
